# Development and validation of a short version of the Sarcopenia Quality of Life questionnaire: the SF-SarQoL

**DOI:** 10.1007/s11136-021-02823-3

**Published:** 2021-03-30

**Authors:** A. Geerinck, C. Beaudart, J.-Y. Reginster, M. Locquet, C. Monseur, S. Gillain, O. Bruyère

**Affiliations:** 1 Division of Public Health, Epidemiology and Health Economics, World Health Organization Collaborating Centre for Public Health Aspects of Musculoskeletal Health and Ageing, University of Liège, Liège, Belgium; 2 Chair for Biomarkers of Chronic Diseases, Biochemistry Department, College of Science, King Saud University, Riyadh, Kingdom of Saudi Arabia; 3 Department of Education Sciences, University of Liège, Liège, Belgium; 4 Geriatrics Department, University Hospital of Liège, Liège, Belgium

**Keywords:** Sarcopenia, Quality of life, Questionnaire development, Item response theory, Item reduction

## Abstract

**Purpose:**

To facilitate the measurement of quality of life in sarcopenia, we set out to reduce the number of items in the previously validated Sarcopenia Quality of Life (SarQoL^®^) questionnaire, and to evaluate the clinimetric properties of this new short form.

**Methods:**

The item reduction process was carried out in two phases. First, information was gathered through item-impact scores from older people (*n* = 1950), a Delphi method with sarcopenia experts, and previously published clinimetric data. In the second phase, this information was presented to an expert panel that decided which of the items to include in the short form. The newly created SFSarQoL was then administered to older, community-dwelling participants who previously participated in the SarcoPhAge study. We examined discriminative power, internal consistency, construct validity, test–retest reliability, structural validity and examined item parameters with a graded response model (IRT).

**Results:**

The questionnaire was reduced from 55 to 14 items, a 75% reduction. A total of 214 older, community-dwelling people were recruited for the validation study. The clinimetric evaluation showed that the SF-SarQoL^®^ can discriminate on sarcopenia status [EWGSOP2 criteria; 34.52 (18.59–43.45) vs. 42.86 (26.56–63.69); *p *= 0.043], is internally consistent (*α* = 0.915, *ω* = 0.917) and reliable [ICC = 0.912 (0.847–0.942)]. A unidimensional model was fitted (CFI = 0.978; TLI = 0.975; RMSEA = 0.108, 90% CI 0.094–0.123; SRMR = 0.055) with no misfitting items and good response category separation.

**Conclusions:**

A new, 14-item, short form version of the Sarcopenia Quality of Life questionnaire has been developed and shows good clinimetric properties.

**Supplementary Information:**

The online version contains supplementary material available at 10.1007/s11136-021-02823-3.

## Background

The process of ageing is associated with numerous physiological changes. One of these changes is the age-related decrease in muscle mass and function known as sarcopenia, which has received a great deal of interest in the past decade [1, 2].

Sarcopenia is described by the European Working Group on Sarcopenia in Older People (EWGSOP) as “*a progressive and generalized skeletal muscle disorder that is associated with increased likelihood of adverse outcomes including falls, fractures, physical disability and mortality*” [3]. The most recent consensus criteria of the EWGSOP2 state that low muscle strength is an indicator of probable sarcopenia, low strength in combination with low muscle mass is confirmed sarcopenia, and low muscle strength, low muscle mass and low physical performance is severe sarcopenia [3].

Sarcopenia has been associated with increased mortality, functional decline, a higher rate of falls and a higher incidence of hospitalization [4, 5]. In the last few years, evidence has been accumulating on the adverse impact of sarcopenia on quality of life [6, 7].

In 2015, Beaudart and colleagues presented the Sarcopenia Quality of Life (SarQoL) questionnaire, an auto-administered patient-reported outcome measure specifically designed to measure quality of life in older, community-dwelling people [8]. It is still currently the only instrument measuring quality of life validated for sarcopenic samples and the only sarcopenia-specific QoL questionnaire available.

The clinimetric properties of the SarQoL questionnaire have been examined for 11 language-specific versions of the questionnaire and has demonstrated strong measurement properties [9–20]. The questionnaire has been extensively translated, and is available in 30 languages from the website www.sarqol.org.

The comprehensive nature of the SarQoL^®^ questionnaire, which allows it to probe multiple facets of QoL in sarcopenia, means a trade-off has been made between its comprehensiveness and its response burden. Several factors may contribute to the perception of burden on the part of the respondent, such as the length of the questionnaire, the formatting, the instructions, the invasiveness of the questions and the cognitive load the questions put on the respondent [21]. While the developers estimated, based on the results of a pre-test in the target population, that it would take most patients about 10 min to complete the SarQoL^®^, in practice a considerable number of respondents need more time than this. Given that most clinical studies administer a number of tests and questionnaires, and thus need to take into consideration the response burden of each instrument so as not to jeopardize the accuracy of the obtained data and the percentage of missing responses, a shorter version of the SarQoL^®^ questionnaire might prove valuable.

The first objective of this study was to extract a shorter version out of the 55 items of the SarQoL^®^ questionnaire which safeguards the conceptual structure and the content validity of the original instrument. The second objective was to investigate the clinimetric properties of the newly developed short-form SarQoL.

## Methods

### Development phase

#### The SarQoL questionnaire

The short form described in this article was developed from the Sarcopenia Quality of Life (SarQoL) questionnaire. This auto-administered patient-reported outcome measure was developed with the specific aim of evaluating quality of life in sarcopenic, community-dwelling older people. The SarQoL measures QoL through 55 items categorized into seven domains of health-related dysfunction: physical and mental health, locomotion, body composition, functionality, activities of daily living, leisure activities, and fears [8]. The response options of the SarQoL questionnaire are a mix of Likert scales (3, 4, or 5 levels) and multiple-answer multiple-choice questions. The scoring algorithm calculates an overall QoL score which is scaled from 20 to 100 points (with complete data), and also provides seven domain scores, scaled from 0 (worst QoL possible) to 100 (best QoL possible) points. The scoring algorithm is not publicly available, but tools to calculate the scores are available by contacting info@sarqol.org. The clinimetric properties of the questionnaire have been evaluated in 11 different language-specific versions, and considerable information is available for known-groups validity, construct validity, internal consistency, floor and ceiling effects, test–retest reliability, standard error of measurement, smallest detectable change, and an evaluation of the responsiveness of the SarQoL has also been carried out [9–20]. Based on these results, the SarQoL is considered to be a valid, reliable and responsive instrument. The SarQoL questionnaire itself and additional information on the various publications are available from www.sarqol.org.

#### Item selection process

The objectives of the item reduction process were to create a significantly shorter version of the SarQoL questionnaire that would represent as much of the conceptual model of the Overall QoL score of the original questionnaire as possible, and thus also be highly correlated with the same score.

**Fig. 1:**
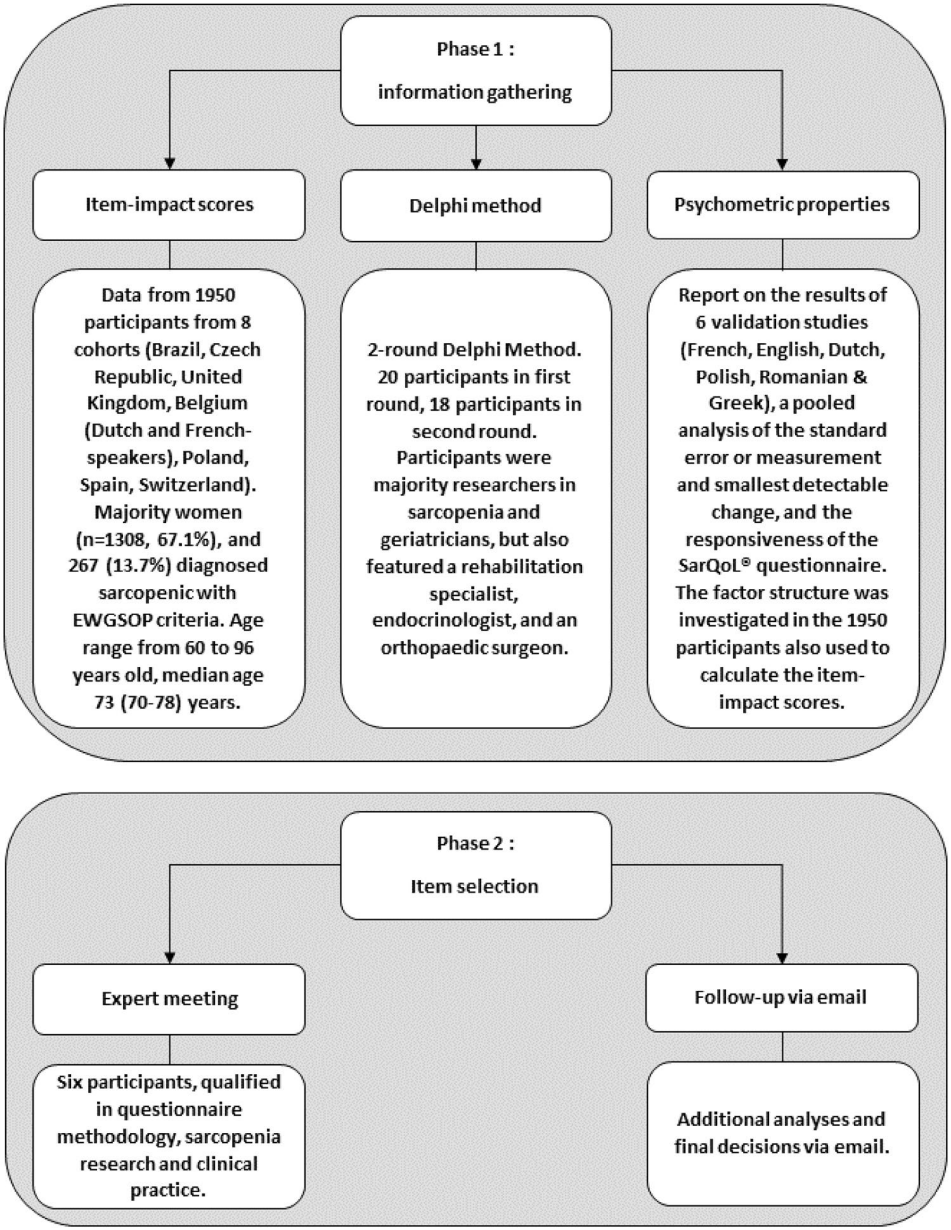
Development of the SF-SarQoL

The item selection process was carried out in two phases, presented in Fig. 1. The first phase served to collect and collate as much information on the properties of the items and domains in the SarQoL questionnaire. This phase started off with the calculation of item-impact scores to determine which items in the SarQoL questionnaire are the most relevant and impactful for sarcopenic people. For this purpose, we combined data collected in Brazil, the Czech Republic, the UK, Belgium (two separate cohorts), Poland, Spain and Switzerland. All data were collected in non-interventional studies (transversal and cohort) from community-dwelling older people (60 years and older) who were evaluated for sarcopenia according to the EWGSOP criteria [22]. In total, data from 1950 participants were included in this dataset, of which 267 were diagnosed as sarcopenic. By calculating the prevalence of an item occurring (those that experienced an item divided by those that did not) and dividing this by the mean impact, a ranking was established from most relevant and impactful to least [23]. The first phase of the item selection process continued with a 2-round modified Delphi method, so that the patient’s perspective quantified by the item-impact scores could be complemented with the opinion of health care professionals and researchers. We targeted researchers and clinicians involved in sarcopenia research who had previous experience with the SarQoL questionnaire, through use, translation, validation or development, and invited them to participate. The participants were provided with an Excel file wherein they were able to categorize each of the 55 items as either “must absolutely be kept in a short form” or “could be discarded”. Items were organized and presented per domain. In the second round, the participants were once again asked to categorize the items in the SarQoL questionnaire (keep or discard), but were now also provided the item-impact scores as well as the percentage of participants who agreed on whether to keep or discard an item in the first round. Consensus at the end of the second round was defined as 70% agreement. During both rounds, participants were able to add comments on their choices. The information from the Delphi method, the item-impact scores, and the already published information concerning the clinimetric properties of the SarQoL questionnaire was summarized into a report at the end of the first stage.

In the second phase of the item reduction process the report compiled at the end of phase one was presented to an expert group consisting of researchers specialized in sarcopenia and QoL, a clinical practitioner and a questionnaire methodologist (AG, CB, OB, ML, CM, SG). These discussed the available information and decided on the inclusion or exclusion of a number of items. As recommended in the guidelines formulated by Goetz et al., the expert group was asked to consider content validity (i.e. the results from the item-impact study and the Delphi method) as having the most weight in the decision-making process, followed by clinimetric properties and finally any additional analyses (factor analysis, correlations, or subgroup analyses) that were performed. To ensure an important reduction of the length of the questionnaire, an arbitrary goal of at least a 65% reduction was chosen at the start of the selection process, while maintaining the relative weight of the seven domains in the SarQoL questionnaire.

### Validation phase

#### Population and study design

For the validation of the SF-SarQoL, we contacted the 314 participants who had previously participated in the fourth and/or fifth year of follow-up of the SarcoPhAge (Sarcopenia and Physical impairment with advancing Age) study [24]. In short, this study recruited older, community-dwelling volunteers from the Liège province of Belgium, and invited them once a year for a battery of physical and other measurements. Given that sarcopenia was the main focus of the SarcoPhAge study, body composition, muscle strength and physical performance were evaluated at each visit with dual-energy X-ray absorptiometry, a hydraulic hand-dynamometer and the Short Physical Performance Battery. Details on the SarcoPhAge study design and results have been reported previously [24, 25]

We provided the participants, through the postal service, with study packets composed of the short form SarQoL questionnaire, the EQ-5D and EQ-VAS questionnaire which are preference-based measures of health status, and the original SarQoL questionnaire. The study packets were accompanied by an explanatory letter and a pre-stamped envelope with which to return the study documents [26]. The people who consented to participate and sent back the completed questionnaires received a second packet by mail about 10 days after the date on which they completed the first packet. The second study packet consisted of the SF-SarQoL and a query on whether their health had changed in the interval between the two administrations of the SF-SarQoL. Demographic and clinical data were obtained from the existing datasets collected during the fourth or fifth year follow-up visits of the SarcoPhAge study. Sarcopenia was diagnosed with the revised consensus criteria from the EWGSOP2 (handgrip strength below 27 kg for men or 16 kg for women, together with low muscle mass defined as appendicular skeletal muscle mass divided by height-squared (ASM/Ht^2^) < 7.0 kg/m^2^ for men or < 5.5 kg/m^2^ for women) [3]. The research protocol (no 2012/277) and its amendment (dated 19/12/2019) were approved by the Ethics Committee of the University Teaching Hospital of Liège.

#### Clinimetric properties from classical test theory

The clinimetric properties of the SF-SarQoL have been examined with the following indicators from classical test theory:

1. Item characteristics have been evaluated with percentage of missing responses. Floor and ceiling effects for the overall QoL score of the SF-SarQoL were considered to be present if more than 15% of respondents obtained the lowest (0 points) or highest (100 points) score [27].
2. Discriminative power (also known as known-groups validity), which measures an instrument’s ability to distinguish among distinct groups, has been examined in three separate comparisons: sarcopenic versus non-sarcopenic, probably sarcopenic (low grip strength in the EWGSOP2 algorithm) versus probably non-sarcopenic (normal grip strength), and at high risk of sarcopenia (SARC-F score ≥ 4) versus at low risk of sarcopenia [3, 28]. We expected to find significantly lower QoL scores on the SF-SarQoL for sarcopenic participants, those with low grip strength and those at high risk of sarcopenia. Significant differences in QoL were established with the Student *t* test or the Mann–Whitney *U* test, depending on normality of distribution of the scores. Point biserial correlation coefficients (*r*) were calculated to provide a measure of the strength of association between group status and QoL.
3. Internal consistency was measured with both the Cronbach’s alpha value and the McDonald omega value. We decided on this approach because the alpha value allows comparison to previous validation studies, while the omega value avoids some of the problems associated with the alpha value and is considered to be a more accurate reflection of internal consistency [29]. For both indicators, values between 0.7 and 0.95 indicate that the items in the questionnaire are closely interrelated and measure the same concept [27].
4. Test–retest reliability has been quantified with the intraclass correlation coefficient (ICC—two-way mixed model and absolute agreement type) for the total score of the SF-SarQoL, and with weighted kappa coefficients (using quadratic weights) for the individual items. An ICC value greater than 0.7 indicates acceptable reliability [27]. For the weighted kappa coefficients, a value ≥ 0.8 is almost-perfect agreement, ≥ 0.6 and < 0.8 is substantial agreement, ≥ 0.4 and < 0.6 is moderate agreement, ≥ 0.2 and < 0.4 is fair agreement and < 0.2 is slight agreement [30]. Only those participants who participated in both administrations of the SF-SarQoL, whose health did not change in the interval period, and who completed the second questionnaire a maximum of 3 weeks after the first, were eligible for inclusion in this analysis. A Bland–Altman analysis was also carried out to detect whether there was systematic bias in the test–retest data [31].
5. The construct validity of the SF-SarQoL has been investigated through three approaches. First, we evaluated criterion validity, where the instrument scores are compared to those of a gold standard. This was measured with the ICC (two-way mixed model and consistency type) between the overall QoL scores of the short form and the original SarQoL questionnaire [27]. Secondly, we tested hypotheses on the expected correlation between the SF-SarQoL and the EQ-5D and EQ-VAS questionnaires, assuming that we will find strong correlations between them [27]. Lastly, we evaluated the structural validity of the SF-SarQoL. We hypothesized that the SF-SarQoL is unidimensional, with all items loading on the latent construct of quality of life, and have carried out a confirmatory factor analysis using the diagonally weighted least squares estimator (WSLMV) for ordinal data using the R package “Lavaan” (version 0.6–6). Model fit was evaluated with the Chi-square test (*p *≥ 0.05 indicates good fit), the comparative fit index (CFI; good fit if ≥ 0.95), the Tucker-Lewis index (TLI; good fit if ≥ 0.95), the root mean square error of approximation (RMSEA; good fit if ≤ 0.08) and the standardized root mean square residual (SRMR; good fit if ≤ 0.08) [32, 33].

#### Clinimetric properties from modern measurement theory

Before constructing and testing an IRT model, it is important to verify that the items meet the assumptions of unidimensionality, local independence and monotonicity [34].

1. Most IRT applications require a factor structure with a single latent trait, hence the need to establish whether the instrument in question is unidimensional. This was established using the results of the CFA described in the previous paragraph, supplemented with an exploratory factor analysis. Before launching the EFA, we inspected the suitability of the data using Bartlett’s test of sphericity and the Kaiser–Meyer–Olkin (KMO) measure of sampling adequacy. The EFA was executed on the polychoric correlation matrix with the WLSMV estimator from the R package “Psych” (version 1.9.12.31). The number of factors present was evaluated with parallel analysis (PA) and Velicer’s minimum average partial (MAP) test [35].
2. The second assumption, local independence, means that there should be no correlation between two items after the effect of the underlying trait is filtered out. In other words, the item responses should be entirely a function of the underlying trait, and not (partly) dependent on a second factor [34]. To determine this, we looked at the residual correlation matrix from the previously described single-factor CFA, and considered a value of 0.2 above the average residual correlation as the cut-off for local independence [36].
3. Lastly, the concept of monotonicity was examined. This concept states that the probability of endorsing a higher item response category should increase with increasing levels of the underlying construct [34]. Monotonicity was evaluated with Mokken scaling carried out with the R package “Mokken” (version 3.0.2), using the scalability coefficient *H* for each item and the questionnaire in its entirety. The assumption of monotonicity was confirmed if the item scalability coefficients were ≥ 0.3 and the scalability coefficient *H*_*i*_ for the entire questionnaire was ≥ 0.5 [36].

After confirming unidimensionality, local independence and monotonicity, a logistic Graded Response Model (GRM) was fit to the data using the R package “mirt” (version 1.32.1). This model calculates both item thresholds (b) as well as item slopes (a). For the purpose of this analysis, the response options “I do not undertake these types of physical activities” in item 2.1 and 2.2, “not applicable” in item 3.1 and 3.2, “I am unable to walk” in item 4, and “I have never participated in leisure activities” in item 8 were treated as missing responses. The encoding of the responses on item 8 was also re-ordered, going from decreased participation to increased participation. Item fit was examined with the *S* − *X*^2^ indicator, where *p *≤ 0.001 indicates poor fit, and by examining the category characteristic curves. For all items, 3 thresholds were estimated, except for item 8, where only two thresholds were estimated.

#### Statistical analysis

All analyses were executed with SPSS version 27.0.0, R version 4.0.0. and JASP version 0.13.1.

In addition to the statistical manipulations described in the preceding paragraphs, we also verified normality of distribution for quantitative variables with the Shapiro–Wilk test, by comparing mean and median, and by evaluating the histogram and Q–Q plot. Continuous variables following a Gaussian distribution are reported as mean ± standard deviation, while skewed variables are reported as median (25th percentile–75th percentile). Nominal variables are reported as absolute (*n*) and relative (%) frequencies. All comparisons were considered significant at the 5% level (*p *≤ 0.5).

## Results

### Development

Twenty experts participated in the first round of the modified Delphi method, and eighteen of them participated in both rounds. The panel reached consensus on the inclusion of 13 items and the exclusion of 23 items, with 19 items not reaching the 70% agreement threshold for either option. Together with the item-impact scores, calculated separately for the sarcopenic (*n* = 267) and non-sarcopenic (*n* = 1584) participants, and the clinimetric information already available from previous validation studies, these allowed the expert panel to reach a final decision on the inclusion of 14 items from six domains (physical and mental health, locomotion, body composition, functionality, activities of daily living, and leisure activities), which together constitute the short-form SarQoL questionnaire. The expert panel made the decision to deviate from the original conceptual model by not including an item from domain seven (fears) because the format of the question ( items are conditional upon the previous question) and the response options (only a positive answer is identified, a negative response or missing data cannot be separated) rendered item-level analysis problematic. The summarized results from the Delphi method, the item-impact ranking and the final decisions of the expert panel are shown in Table 1. The SF-SarQoL is available in online supplementary 1 and from www.sarqol.org.

**Table 1:** Development SF-SarQoL

Domain/item		Delphi method^a^		Item-impact ranking^b,c^		Final decision
		Consensus inclusion	Consensus exclusion	Sarcopenic group	Non-sarcopenic group	
Physical and mental health						
1.1 Loss of arm strength		x		3	6	IN
1.2 Loss of leg strength		x		1	4	IN
1.4 Loss of energy			x	4	5	
2 Muscle pain			x	2	3	
6 Feeling old			x	6	2	
7 Feeling of muscle weakness			x			
8 Feeling of being physically weak				5	1	IN
16 Feeling of being frail				7	7	
Locomotion						
9.1 Limitation in walking time		x		4	4	
9.2 Limitation in number of outings			x	6	6	
9.3 Limitation in walking distance		x		2	2	
9.4 Limitation in walking speed		x		1	1	IN
9.5 Limitation in steps length			x	7	7	
10.1 Feeling of fatigue when walking		x		3	3	IN
10.2 Need of recovery time when walking				7	8	
10.3 Difficulties to cross a road fast enough				9	9	
10.4 Difficulties to walk on uneven ground			x	4	5	
Body composition						
1.3 Loss of muscle mass				2	2	IN
13 Physical change			x	1	1	
14 Weight change (loss or gain)			x			
15 Upset with change			x			
Functionality						
1.5 Loss of physical capacity		x		2	2	IN
1.6 Loss of flexibility			x	3	1	
11 Balance problems				5	4	IN
12 Falls occurrence		x		13	8	
17.1 Climbing one flight of stairs		x		11	13	
17.2 Climbing several flights of stairs			x	6	6	
17.3 Climbing stairs without a banister				8	11	
17.4 Crouching or kneeling				4	5	
17.5 Stooping				10	10	
17.6 To stand up from the floor without any support				1	3	IN
17.7 Get up from a chair		x		9	7	
17.8 To stand from a sitting position				12	12	
18 Limitation of movement		x		7	9	IN
20 Sexuality			x	14	14	
Activities of daily living						
17.11 Take public transportation			x	14	14	
17.12 To get in/out a car			x	12	12	
3.1 Difficulty during light physical effort				5	9	
3.2 Fatigue during light physical effort		x		2	7	
3.3 Pain during light physical effort			x	4	8	
4.1 Difficulty during moderate physical effort				6	6	IN
4.2 Fatigue during moderate physical effort		x		3	3	IN
4.3 Pain during moderate physical effort			x	7	5	
5.1 Difficulty during intense physical effort			x	10	2	
5.2 Fatigue during intense physical effort			x	9	1	
5.3 Pain during intense physical effort			x	11	4	
17.9 Carrying heavy objects			x	1	10	IN
17.10 Opening a bottle or a jar				8	11	
17.13 Shopping			x	15	15	
17.14 Household tasks				13	13	
Leisure activities						
21 Change in physical activities			x	1	1	
22 Change in leisure activities				2	2	IN
Fears						
19	Fear of getting hurt Fear of not succeeding Fear of being tired Fear of falling					

^a^Empty cells indicate that the 70% agreement threshold was not reached

^b^Because certain questions in the SarQoL questionnaire are conditional on other questions (i.e. “If yes on previous question, then …”), item-impact scores could not be calculated for items 7, 14, 15 and 19

^c^Items are ranked from most impactful (1) to least impactful

### Clinimetric evaluation

#### Participants

A total of 214 older people participated in the validation study for the SF-SarQoL. The median age of the participants was 76 (73–81) years and 63.1% were women. We found 70 (32.7%) participants with probable sarcopenia (low grip strength in the EWGSOP2 algorithm), of whom 21 (9.8%) had confirmed sarcopenia. With the help of the SARC-F questionnaire, we found 30 (14.0%) participants at high risk of sarcopenia. The complete clinical and QoL characteristics are reported in Table 2.

**Table 2:** Characteristics of the sample

	*n* (%)	Median (P25–P75)
Gender		
Male	80 (36.9%)	
Female	137 (63.1%)	
Age (years)		76 (73–81)
Probable sarcopenia (with EWGSOP2)		
Yes	70 (32.7%)	
No	143 (66.8%)	
Sarcopenia (with EWGSOP2)		
Yes	21 (9.8%)	
No	193 (90.2%)	
At risk of sarcopenia (with SARC-F)		
Yes	30 (14.0%)	
No	184 (86.0%)	
EQ-5D index score		0.800 (0.747–0.827)
EQ-VAS		70 (60–80)
SarQoL		
Physical and mental health		60.54 (48.87–73.30)
Locomotion		55.56 (41.67–75.70)
Body composition		62.50 (48.96–70.83)
Functionality		66.69 (55.36–82.28)
Activities of daily living		60.00 (48.21–76.67)
Leisure activities		33.25 (33.25–66.50)
Fears		87.50 (75.00–100.00)
Overall QoL score		61.97 (51.57–75.64)
SF-SarQoL overall QoL score first administration		40.24 (23.81–62.64)
SF-SarQoL overall QoL score second administration		47.62 (31.55–70.24)

#### Relationship between short and long form scoring algorithm

**Fig. 2:**
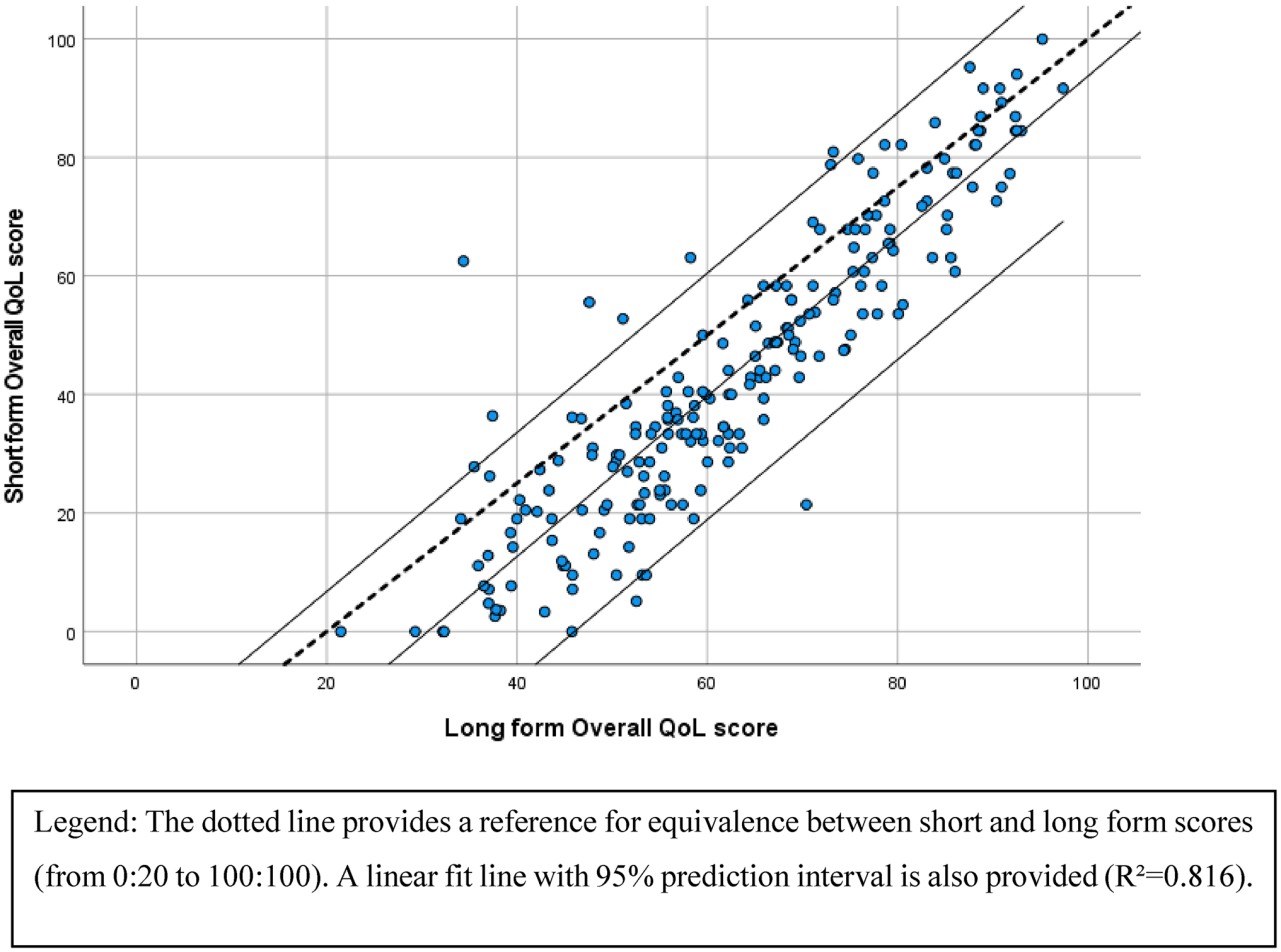
Relation between short form scores and the long form Overall QoL scores

To ease interpretation of the QoL scores of the short form questionnaire, it was decided to use a scale going from zero to 100, a deviation from the 20–100 scale of the long form questionnaire. Within the scale, lower scores represent persons whose quality of life is significantly impacted by sarcopenia, and higher scores indicate people with better QoL and a smaller impact of sarcopenia. Figure 2 shows the scatter plot of the short and long form Overall QoL score. From this figure, it can be observed that the short form scores Overall QoL scores are roughly parallel but below the dotted equivalence line, which represents perfect correspondence between the 2 scores.

#### Clinimetric properties classical test theory

The per-item percentage of missing responses ranged between 0 and 5.6%. Five (2.3%) participants scored zero points on the Overall QoL score of the SF-SarQoL, and 1 (0.5%) person scored 100 points, indicating that there are no floor or ceiling effects in this sample. We found excellent discriminative power when comparing probably sarcopenic versus probably not [32.74 (20.15–43.15) vs. 48.81 (28.57–70.24); *p *< 0.001; *r* = − 0.342], sarcopenic versus not sarcopenic [34.52 (18.59–43.45) vs. 42.86 (26.56–63.69); *p *= 0.043; *r* = − 0.144] and at high risk of sarcopenia versus low risk [17.86 (6.64–24.05) vs. 46.43 (30.95–65.48); *p *< 0.001; *r* = − 0.444]. Internal consistency among the items was excellent with a Cronbach’s alpha of 0.915 (95% CI = 0.896–0.930) and a McDonalds’ omega value of 0.917 (95% CI = 0.897–0.933). Test–retest reliability was calculated among 133 participants. Within this sub-sample, we found excellent test–retest reliability with an ICC of 0.912 (95% CI = 0.847–0.942) for the overall QoL score of the SF-SarQoL. On an item level, we found moderate to almost-perfect agreement between the first and second administration with weighted kappa coefficients, detailed in Table 3.

**Table 3:** Test–retest reliability and construct validity

	Concordance of items between test and retest (*n* = 133)		Standardized factor loadings		
	Weighted kappa (95% CI)	Interpretation^a^	Model 1	Model 2^b^	
				Factor 1	Factor 2
1.1 Reduction strength arms	0.794 (0.658–0.840)	Substantial	0.695	0.725	
1.2 Reduction strength legs	0.735 (0.637–0.834)	Substantial	0.897	0.930	
1.3 Reduction muscle mass	0.682 (0.590–0.773)	Substantial	0.806	0.827	
1.4 Reduction physical capacity	0.613 (0.495–0.732)	Substantial	0.917	0.951	
1.5 Reduction length of walks	0.750 (0.673–0.828)	Substantial	0.867	0.873	
2.1 Difficulty moderate effort	0.691 (0.541–0.842)	Substantial	0.901		0.915
2.2 Tired moderate effort	0.646 (0.485–0.808)	Substantial	0.856		0.864
3.1 Get up from floor	0.683 (0.512–0.854)	Substantial	0.786		0.802
3.2 Carrying heavy objects	0.546 (0.335–0.756)	Moderate	0.821		0.833
4 Tired when walking	0.798 (0.732–0.865)	Substantial	0.874		0.866
5 Feel weak	0.791 (0.709–0.873)	Substantial	0.877		0.900
6 Balance problems	0.867 (0.812–0.921)	Almost perfect	0.673		0.689
7 Limit movements	0.728 (0.637–0.819)	Substantial	0.850		0.868
8 Leisure activities	0.406 (0.185–0.627)	Moderate	0.594		0.605

^a^Kappas interpreted according to Landis and Koch, where ≥ 0.8 is almost-perfect agreement, ≥ 0.6 and < 0.8 is substantial agreement, ≥ 0.4 and < 0.6 is moderate agreement, ≥ 0.2 and < 0.4 is fair agreement, and < 0.2 is slight agreement

^b^Model 2 is a 2-factor model with correlated residual variance between items 1.5 and 4

A Bland–Altman analysis revealed the presence of a systematic bias of 4.11 (95% CI 2.51; 5.72) points, with higher average scores for the retest scores (50.47 ± 24.82) compared to the test scores (46.36 ± 23.30).

The criterion construct validity, measuring the strength of relationship between the SarQoL overall QoL score and its short form equivalent, was excellent with an ICC of 0.835 (95% CI = 0.789–0.871). It should be noted that the scoring algorithm for the short form and the original SarQoL questionnaire are not on the same metric, and are thus not interchangeable. We also found strong correlations between the SF-SarQoL overall score and the EQ-5D index score (*r* = 0.671; *p *< 0.001) and the EQ-VAS (*r* = 0.697; *p *< 0.001). A confirmatory factor analysis of a one-dimensional model resulted in the following fit indices (*χ*^2^ = 269.330, df = 77, *p *< 0.001; CFI = 0.978; TLI = 0.975; RMSEA = 0.108, 90% CI = 0.094–0.123; SRMR = 0.055). As the five items of question 1 share a common stem, we hypothesized that they would be highly correlated, with would lead to a deterioration of fit indices. To overcome this issue, an alternative model was tested, with the five items of question 1 loading on a first latent variable, and the remaining questions on a second latent variable (factor 1: items 1.1 to 1.5; factor 2: items 2.1 to 8) and a correlated residual variance between items 1.5 and 4. This model obtained adequate fit indices: (*χ*^2^ = 161.847, df = 75, *p *< 0.001; CFI = 0.990; TLI = 0.988; RMSEA = 0.074, 90% CI = 0.058–0.089; SRMR = 0.042). The 2 latent variables in this model are highly correlated at *r* = 0.894. Standardized factor loadings for both models are reported in Table 3.

**Table 4:** Graded response model

Item	Monotonicity	Model fit	Item parameters			
	*H*_*i*_	*p* value * S* − *X*^2a^	*a*	*b*_1_	*b*_2_	*b*_3_
1.1 Reduction strength arms	0.526	0.061	1.691	− 1.519	0.277	1.579
1.2 Reduction strength legs	0.681	0.407	3.515	− 0.618	0.314	1.388
1.3 Reduction muscle mass	0.590	0.460	2.278	− 1.140	0.292	1.499
1.4 Reduction physical capacity	0.716	0.365	3.791	− 1.012	0.494	1.594
1.5 Reduction length of walks	0.653	0.204	2.940	− 0.543	0.415	1.461
2.1 Difficulty moderate effort	0.695	0.176	3.592	− 0.478	0.361	1.262
2.2 Tired moderate effort	0.651	0.072	2.790	− 0.416	0.618	1.756
3.1 Get up from floor	0.591	0.001	2.219	− 1.002	0.193	1.378
3.2 Carrying heavy objects	0.653	0.497	2.544	− 1.889	− 0.314	1.012
4 Tired when walking	0.645	0.068	3.176	− 0.673	0.247	1.425
5 Feel weak	0.687	0.476	3.386	− 0.967	0.234	1.210
6 Balance problems	0.517	0.632	1.581	− 1.362	0.022	1.298
7 Limit movements	0.697	0.269	2.954	− 1.335	− 0.110	0.709
8 Leisure activities	0.557	0.435	1.518	0.017	3.229	NA

^a^*S* − *X*^2^ statistic calculated on 160 complete observations

#### Clinimetric properties modern measurement theory

Confirmatory factor analysis did not conclusively indicate that the SF-SarQoL is unidimensional. Therefore, we investigated further with an exploratory factor analysis, which was considered appropriate when the Bartlett’s test returned a *p* value < 0.001 and the KMO test a value of 0.87. Parallel analysis identified a single factor in the data, as did the Velicer’s MAP test, which achieved a minimum of 0.05 with 1 factor. There were no locally dependent items found, with no residual correlations greater than the cut-off of 0.184 or − 0.216 (average residual correlation = − 0.016). The monotonicity assumption was confirmed when scalability coefficients *H*_*i*_ between 0.517 (“balance problems”) and 0.716 (“reduction physical capacity”) were found, alongside a Mokken scalability coefficient *H* for the entire short form of 0.635.

**Fig. 3:**
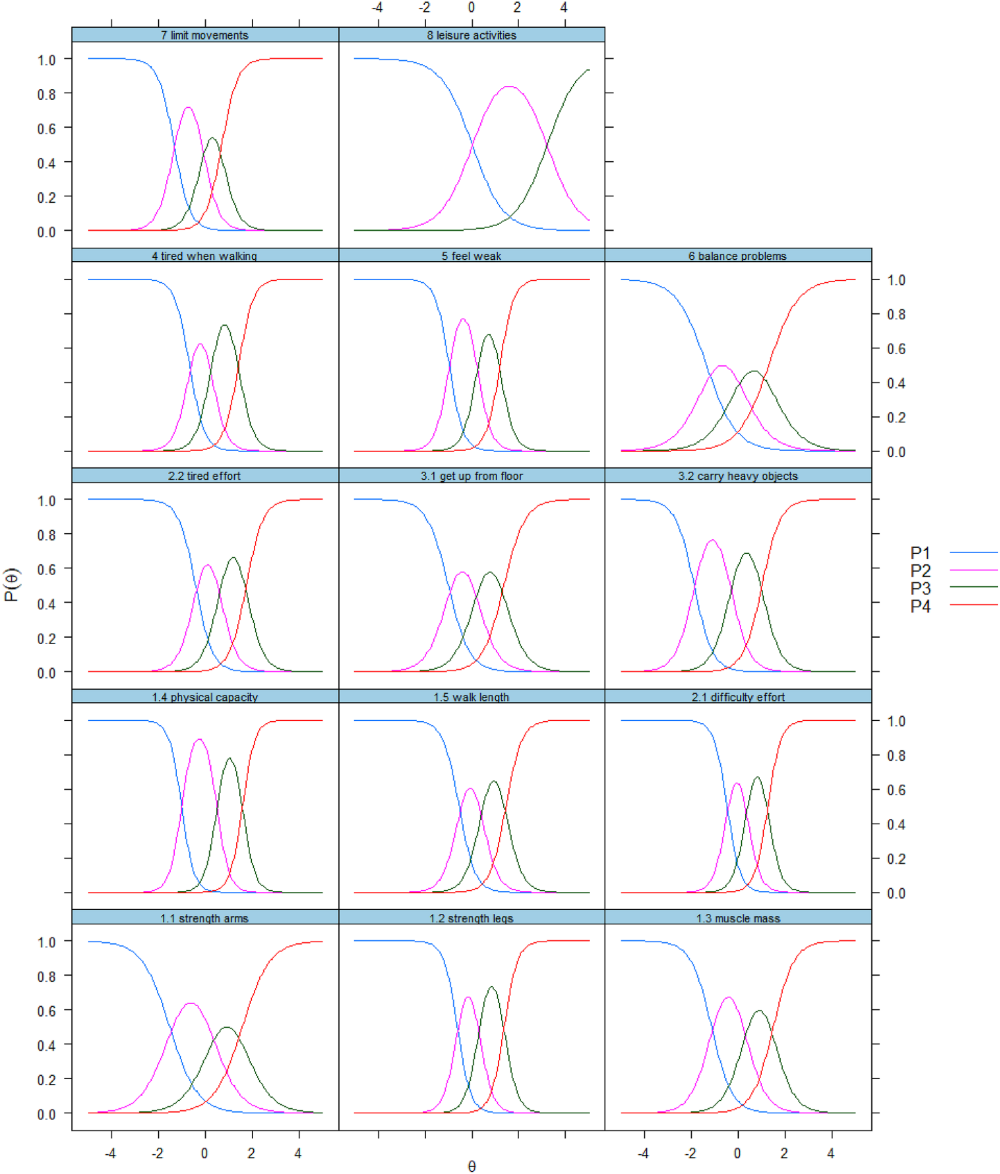
Category characteristic curves of the 14 items analyzed in the SF-SarQoL

After fitting the logistic Graded Response Model to the data, we found no misfitting items, as evidenced by the fact that no *p* values for the *S* − *X*^2^ indicator were smaller than 0.001. The item with the lowest discriminative ability was found to be “leisure activities” (*a* = 1.518) and the most discriminative item was “reduction of physical capacity” (*a* = 3.791). The item thresholds were spread out from − 1.889 (“Carrying heavy objects”) to 1.756 (“Tired moderate effort”). Detailed results on the model fit and item parameters are reported in Table 4. The category characteristics curves, a visual representation of the item parameters, are shown in Fig. 3.

## Discussion

This article describes the development of a 14-item short form version of the SarQoL^®^ questionnaire, and the subsequent examination of its clinimetric properties.

The item reduction process follows the guidelines formulated by Goetz et al. by, among other things, prioritizing content validity over statistical properties [37]. The 2-phase process employed led to the inclusion of 14 items from six domains, preserving, as much as possible, the conceptual structure of the original SarQoL^®^ questionnaire in the short form. One domain (D7: fears) did not contribute to the short form because, in the original questionnaire, it is dependent on the response of a different item that is not a part of domain seven. This type of conditional question (“If yes to the previous question, then …) combined with the fact that the response options for the items in question 19 make it impossible to distinguish between missing data and negative responses, made it inopportune in the eyes of the expert committee to include this domain. On top of the problems caused by its phrasing and response option, the participants in the Delphi method did not reach consensus on its inclusion, so these items and domain was not included in the short form. The questionnaire was thus reduced from 55 to 14 items, a 75% reduction.

In contrast with the original questionnaire, the newly created SF-SarQoL does not provide domain scores, but only an Overall QoL score. This is a conscious choice because, in our estimation, the original SarQoL^®^ questionnaire is better suited when researchers wish to look at QoL on a domain-level. The SF-SarQoL is better suited to studies that use QoL as a secondary outcome, or in association with a general QoL instrument, and, in this vein, it privileges a single QoL score.

The validation part of this study found good to excellent results for discriminative power, construct validity, internal consistency, test–retest reliability and an absence of floor and ceiling effects. However, despite an ICC of 0.912 (95% CI = 0.847–0.942) for the test–retest reliability, we did find a systematic bias of 4.11 (95% CI = 2.51; 5.72) points. An earlier analysis of the original SarQoL^®^ questionnaire in a sample of 274 sarcopenic participants demonstrated no such bias [0.18 (− 0.26; 0.63) points], so this result was unexpected [11]. It is unclear how this bias originated and whether it is a feature of the questionnaire or a one-off event, specific to this sample. It is possible that the higher QoL scores recorded during the second administration of the SF-SarQoL may be due to the packet length (19 pages for the first packet versus 6 pages for the second packet), or due to the information on sarcopenia received with the first packet, and which was absent in the second packet. Future validation studies should prioritize investigating test–retest reliability and, hopefully, clarify this issue. Confirmatory factor analysis did not conclusively confirm the unidimensional nature of the SF-SarQoL, with a 2-factor model showing better fit than the unidimensional model. The graded response model did not indicate any misfitting items. The item trace lines show good separation between the different response categories.

Overall, the SF-SarQoL displays adequate to good clinimetric properties, allowing its use in research, clinical trials and clinical practice. Potential users should consider the objectives of their research when choosing between the 55-item or the 14-item SarQoL^®^ questionnaire. If QoL is a primary outcome, the original SarQoL^®^ questionnaire provides a superior level of detail and precision, as well as scores for the seven QoL domains on top of the overall QoL score. However, if QoL is not the main objective, and response burden is a serious consideration, the SF-SarQoL could be the more appropriate tool.

An important remark to make is that the scores on the original SarQoL^®^ questionnaire and the newly developed SF-SarQoL are not interchangeable and should not be compared head-to-head. During the discussions on the scoring algorithm to be created for the short form SarQoL questionnaire, we examined the complexities of the original scoring algorithm, and a choice was made to place the SF-SarQoL on a 0–100 scale where the score range for the original SarQoL^®^ questionnaire is about 20–100 points.

This study has several strengths: we followed the guidelines by Goetz et al., prioritized content validity, administered the SF-SarQoL in an independent sample and performed as complete a validation as possible with elements from both classical test theory and modern measurement theory.

However, this study also has some limitations: we did not perform differential item functioning analysis because of concerns about the sample size. We fully intend to rectify this once we are able to assemble sufficient data, preferably from multiple countries. We were unable to integrate the domain “fears” into the short-form, so a certain amount of content was lost during the item reduction process. Our sample size of 214 participants is sufficient for the performed statistical manipulations, but does not permit subgroup analyses. The members of the Delphi panel were selected for their previous knowledge of the SarQoL^®^ questionnaire, and were not necessarily representative of the wider community of sarcopenia researchers and geriatricians. Due to the transversal nature of the performed validation study, we were unable to examine the responsiveness of the new SF-SarQoL. Evaluating this property of the SF-SarQoL should be a priority for future research.

In conclusion, this article presented the development process and the validation of a 14-item short form version of the SarQoL^®^ questionnaire. In an independent sample, the SF-SarQoL demonstrated adequate measurement properties to allow its use. While its responsiveness should still be investigated, we fully recommend its use in situations where the original 55-item SarQoL^®^ questionnaire is deemed to be too much of a burden on the respondents.

## Supplementary Information

Below is the link to the electronic supplementary material.

Supplementary file1 (PDF 406 kb)

## Data Availability

The dataset has been deposited on the Open Science Network (OSF) and can be consulted via the following link: http://www.doi.org/10.17605/OSF.IO/3PSZM

## Code availability

R scripts have been deposited on the Open Science Network (OSF) and can be consulted via the following link: http://www.doi.org/10.17605/OSF.IO/3PSZM

## References

[1] Peña Ordóñez GG, Bustamante Montes LP, Ramírez Duran N, Sánchez Castellano C, Cruz-Jentoft AJ (2017). Populations and outcome measures used in ongoing research in sarcopenia. Aging Clinical and Experimental Research.

[2] Reginster JY, Beaudart C, Al-Daghri N, Avouac B, Bauer J, Bere N (2020). Update on the ESCEO recommendation for the conduct of clinical trials for drugs aiming at the treatment of sarcopenia in older adults. Aging Clinical and Experimental Research.

[3] Cruz-Jentoft AJ, Bahat G, Bauer J, Boirie Y, Bruyère O, Cederholm T (2018). Sarcopenia: Revised European consensus on definition and diagnosis. Age and Ageing.

[4] Beaudart C, Zaaria M, Pasleau F, Reginster J-Y, Bruyère O, Stenroth L (2017). Health outcomes of sarcopenia: A systematic review and meta-analysis. PLoS ONE.

[5] Zhao Y, Zhang Y, Hao Q, Ge M, Dong B (2019). Sarcopenia and hospital-related outcomes in the old people: A systematic review and meta-analysis. Aging Clinical and Experimental Research.

[6] Woo T, Yu S, Visvanathan R (2016). Systematic literature review on the relationship between biomarkers of sarcopenia and quality of life in older people. The Journal of Frailty & Aging.

[7] Tsekoura M, Kastrinis A, Katsoulaki M, Billis E, Gliatis J (2017). Sarcopenia and its impact on quality of life. Advances in Experimental Medicine and Biology.

[8] Beaudart C, Biver E, Reginster JY, Rizzoli R, Rolland Y, Bautmans I (2015). Development of a self-administrated quality of life questionnaire for sarcopenia in elderly subjects: The SarQoL. Age and Ageing.

[9] Beaudart C, Biver E, Reginster J-Y, Rizzoli R, Rolland Y, Bautmans I (2017). Validation of the SarQoL, a specific health-related quality of life questionnaire for Sarcopenia. Journal of Cachexia, Sarcopenia and Muscle.

[10] Beaudart C, Edwards M, Moss C, Reginster JY, Moon R, Parsons C (2017). English translation and validation of the SarQoL®, a quality of life questionnaire specific for sarcopenia. Age and Ageing.

[11] Geerinck A, Alekna V, Beaudart C, Bautmans I, Cooper C, De Souza OF (2019). Standard error of measurement and smallest detectable change of the Sarcopenia Quality of Life (SarQoL) questionnaire: An analysis of subjects from 9 validation studies. PLoS ONE.

[12] Erdogan T, Eris S, Avci S, Oren MM, Kucukdagli P, Kilic C (2021). Sarcopenia quality-of-life questionnaire (SarQoL)®: Translation, cross-cultural adaptation and validation in Turkish. Aging Clinical and Experimental Research.

[13] Geerinck A, Scheppers A, Beaudart C, Bruyère O, Vandenbussche W, Bautmans R (2018). Translation and validation of the Dutch SarQoL ®, a quality of life questionnaire specific to sarcopenia. Journal of Musculoskeletal and Neuronal Interactions.

[14] Tsekoura M, Billis E, Gliatis J, Tsepis E, Matzaroglou C, Sakkas GK (2018). Cross cultural adaptation of the Greek sarcopenia quality of life (SarQoL) questionnaire. Disability and Rehabilitation.

[15] Konstantynowicz J, Abramowicz P, Glinkowski W, Taranta E, Marcinowicz L, Dymitrowicz M (2018). Polish validation of the SarQoL®, a Quality of Life Questionnaire specific to sarcopenia. Journal of Clinical Medicine.

[16] Gasparik AI, Mihai G, Beaudart C, Bruyere O, Pop R-M, Reginster J-Y (2018). Correction to: Psychometric performance of the Romanian version of the SarQoL(R), a health-related quality of life questionnaire for sarcopenia. Archives of Osteoporosis.

[17] Alekna V, Kilaite J, Tamulaitiene M, Geerinck A, Mastaviciute A, Bruyère O (2019). Validation of the Lithuanian version of sarcopenia-specific quality of life questionnaire (SarQoL®). European Geriatric Medicine.

[18] Safonova YA, Lesnyak OM, Baranova IA, Suleimanova AK, Zotkin EG (2019). Russian translation and validation of SarQoL®—Quality of life questionnaire for patients with sarcopenia. Nauchno-Prakticheskaya Revmatol.

[19] Fábrega-Cuadros R, Martínez-Amat A, Cruz-Díaz D, Aibar-Almazán A, Hita-Contreras F (2020). Psychometric properties of the Spanish version of the sarcopenia and quality of life, a Quality of Life Questionnaire Specific for Sarcopenia. Calcified Tissue International.

[20] Geerinck A, Bruyère O, Locquet M, Reginster JY, Beaudart C (2018). Evaluation of the responsiveness of the SarQoL® questionnaire, a patient-reported outcome measure specific to sarcopenia. Advances in Therapy.

[21] U.S. Department of Health and Human Services F and DA, Center for Drug Evaluation and Research, Center for Biologics Evaluation and Research, Center for Devices and Radiological Health (2009). Guidance for industry use in medical product development to support labeling claims guidance for industry. Retrieved from https://www.fda.gov/media/77832/download.

[22] Cruz-Jentoft A, Baeyens JP, Bauer J, Boirie Y, Cederholm T, Landi F (2010). Sarcopenia: European consensus on definition and diagnosis. Age and Ageing.

[23] Broder HL, McGrath C, Cisneros GJ (2007). Questionnaire development: Face validity and item impact testing of the child oral health impact profile. Community Dentistry and Oral Epidemiology.

[24] Beaudart C, Reginster JY, Petermans J, Gillain S, Quabron A, Locquet M (2015). Quality of life and physical components linked to sarcopenia: The SarcoPhAge study. Experimental Gerontology.

[25] Locquet M, Beaudart C, Hajaoui M, Petermans J, Reginster JY, Bruyère O (2018). Three-year adverse health consequences of sarcopenia in community-dwelling older adults according to 5 diagnosis definitions. Journal of the American Medical Directors Association.

[26] The EuroQol Group (1990). EuroQol: A new facility for the measurement of health-related quality of life. Health Policy (New York).

[27] de Vet HCW, Terwee CB, Mokkink LB (2011). Measurement in medicine: A practical guide.

[28] Malmstrom TK, Morley JE (2013). SARC-F: A simple questionnaire to rapidly diagnose sarcopenia. Journal of the American Medical Directors Association.

[29] Dunn TJ, Baguley T, Brunsden V (2014). From alpha to omega: A practical solution to the pervasive problem of internal consistency estimation. British Journal of Psychology.

[30] Landis JR, Koch GG (1977). The measurement of observer agreement for categorical data. Biometrics.

[31] Bland JM, Altman DG (1999). Measuring agreement in method comparison studies. Statistical Methods in Medical Research.

[32] Brown T (2015). Confirmatory factor analysis for applied research.

[33] Kline R (2016). Principles and practice of structural equation modeling.

[34] DeMars C (2010). Item response theory.

[35] Howard MC (2016). A review of exploratory factor analysis decisions and overview of current practices: What we are doing and how can we improve?. International Journal of Human Computer Interaction.

[36] Lameijer CM, Van Bruggen SGJ, Haan EJA, Van Deurzen DFP, Van Der Elst K, Stouten V (2020). Graded response model fit, measurement invariance and (comparative) precision of the Dutch-Flemish PROMIS® upper extremity V2.0 item bank in patients with upper extremity disorders. BMC Musculoskeletal Disorders.

[37] Goetz C, Coste J, Lemetayer F, Rat AC, Montel S, Recchia S (2013). Item reduction based on rigorous methodological guidelines is necessary to maintain validity when shortening composite measurement scales. Journal of Clinical Epidemiology.

