# Supplementary material for: Development and validation of a short version of the Sarcopenia Quality of Life questionnaire: the SF-SarQoL
Source: Qual Life Res. 2021 Mar 30;30(8):2349–62. doi: 10.1007/s11136-021-02823-3 (PMC8298237; doi:10.1007/s11136-021-02823-3)
Supplement: Supplementary file 1 — Supplementary file1 (PDF 406 kb) [file 11136_2021_2823_MOESM1_ESM.pdf]

# SarQoL
Sarcopenia and Quality of Life

**Questionnaire I** Time: ±5 min

## Quality of life in sarcopenia

This questionnaire asks about sarcopenia, which is a muscle weakness that comes about with ageing. Sarcopenia can affect your daily life. This survey will enable us to find out if the state of your muscles currently affects your quality of life.

Please choose the most appropriate response for each question. The questionnaire should take you approximately 5 minutes to complete.

### 1. Do you currently feel you have a reduction in:

| | A lot | Some | A little | None |
|---|---|---|---|---|
| The strength in your arms? | ☐ | ☐ | ☐ | ☐ |
| The strength in your legs? | ☐ | ☐ | ☐ | ☐ |
| Your muscle mass? | ☐ | ☐ | ☐ | ☐ |
| Your physical capabilities? | ☐ | ☐ | ☐ | ☐ |
| The distance you can walk? | ☐ | ☐ | ☐ | ☐ |

### 2. When undertaking moderate physical activities (fast walking, cleaning windows, hoovering, washing the car, pulling up weeds in the garden, etc.), do you:

| | Often | Occasionally | Rarely | Never | I do not undertake these types of physical activities |
|---|---|---|---|---|---|
| Have difficulty? | ☐ | ☐ | ☐ | ☐ | ☐ |
| Get tired? | ☐ | ☐ | ☐ | ☐ | ☐ |

### 3. Do you currently have difficulty in undertaking any of the following activities:

| | Unable to do | Great difficulty | A little difficulty | No difficulty | Not applicable |
|---|---|---|---|---|---|
| Getting up from the floor without holding on to anything? | ☐ | ☐ | ☐ | ☐ | ☐ |
| Carrying heavy objects (large bags full of shopping, saucepan filled with water, etc.)? | ☐ | ☐ | ☐ | ☐ | ☐ |

Copyright © - November 2019 – All rights reserved

SarQoL
Sarcopenia and Quality of Life

**4.** When you are walking, do you feel very tired?

| Often | Occasionally | Rarely | Never | I am unable to walk |
|---|---|---|---|---|
| ☐ | ☐ | ☐ | ☐ | ☐ |

**5.** Do you feel physically weak?

| Yes, completely | Yes, somewhat | Yes, a little | No, not at all |
|---|---|---|---|
| ☐ | ☐ | ☐ | ☐ |

**6.** Do you have problems with your balance?

| Often | Occasionally | Rarely | Never |
|---|---|---|---|
| ☐ | ☐ | ☐ | ☐ |

**7.** Does your muscle weakness limit your movement?

| Yes, a lot | Yes, somewhat | Yes, a little | No, not at all |
|---|---|---|---|
| ☐ | ☐ | ☐ | ☐ |

**8.** Has your participation in leisure activities (going out to eat, gardening, doing DIY, shooting/fishing, senior citizens clubs, playing bridge, going for a walk, etc.) changed?

| Increased | Decreased | Unchanged | I have never participated in leisure activities |
|---|---|---|---|
| ☐ | ☐ | ☐ | ☐ |

Copyright © - November 2019 – All rights reserved
